# Effect of Probiotic Supplements on the Oral Microbiota—A Narrative Review

**DOI:** 10.3390/pathogens13050419

**Published:** 2024-05-16

**Authors:** Christine Lundtorp-Olsen, Merete Markvart, Svante Twetman, Daniel Belstrøm

**Affiliations:** Department of Odontology, Section for Clinical Oral Microbiology, Faculty of Health and Medical Sciences, University of Copenhagen, 2200 Copenhagen, Denmark; christine.olsen@sund.ku.dk (C.L.-O.); mema@sund.ku.dk (M.M.); stwe@sund.ku.dk (S.T.)

**Keywords:** probiotics, microbiota, periodontitis, dental caries, gingivitis, oral health

## Abstract

Data from systematic reviews and meta-analyses show that probiotics positively impact clinical parameters of oral diseases such as gingivitis, dental caries, and periodontitis. However, the working mechanism of probiotics is not fully understood, but is hypothesized to be mediated by direct and indirect interactions with the oral microbiota and the human host. In the present narrative review, we focused on the microbiological effect of probiotic supplements based on data retrieved from randomized clinical trials (RCTs). In addition, we assessed to what extent contemporary molecular methods have been employed in clinical trials in the field of oral probiotics. Multiple RCTs have been performed studying the potential effect of probiotics on gingivitis, dental caries, and periodontitis, as evaluated by microbial endpoints. In general, results are conflicting, with some studies reporting a positive effect, whereas others are not able to record any effect. Major differences in terms of study designs and sample size, as well as delivery route, frequency, and duration of probiotic consumption, hamper comparison across studies. In addition, most RCTs have been performed with a limited sample size using relatively simple methods for microbial identification, such as culturing, qPCR, and DNA–DNA checkerboard, while high-throughput methods such as 16S sequencing have only been employed in a few studies. Currently, state-of-the-art molecular methods such as metagenomics, metatranscriptomics, and metaproteomics have not yet been used in RCTs in the field of probiotics. The present narrative review revealed that the effect of probiotic supplements on the oral microbiota remains largely uncovered. One important reason is that most RCTs are performed without studying the microbiological effect. To facilitate future systematic reviews and meta-analyses, an internationally agreed core outcome set for the reporting of microbial endpoints in clinical trials would be desirable. Such a standardized collection of outcomes would most likely improve the quality of probiotic research in the oral context.

## 1. Background

Oral health is shaped by the symbiotic relationship between the oral microbiota and the host [1], with the oral microbiota being critically involved in transitions from oral health towards the three major oral diseases—gingivitis, periodontitis, and dental caries [2,3,4]. Hence, treatment and prevention of oral diseases have traditionally had a strong microbiological focus, targeting oral biofilms [5,6].

Probiotics are defined by the World Health Organization (WHO) as live microorganisms which, when administrated in adequate amounts, confer health benefits to the host [7]. Probiotics, mainly *Lactobacillus* and *Bifidobacterium* species, have been demonstrated to have potential beneficial effects in the gastrointestinal area, including food allergy [8]. While the mode of action of probiotics is not completely understood in detail, one of the main desired actions is antimicrobial activity against pathogenic bacteria [9]. Hence, when considering the global burden of antibiotic resistance [10], probiotics are an attractive antimicrobial alternative to antibiotics.

Probiotics have been extensively tested in prevention and/or treatment of oral diseases, with results being conflicting. For example, two recent meta-analyses from 2022 and 2023 concluded that probiotics influenced clinical periodontal parameters such as bleeding on probing and probing pocket depth [11,12]. On the contrary, two other meta-analyses of ten and four clinical trials, respectively, did not show any effect of probiotics on bleeding on probing in patients with gingivitis [13,14]. In dental caries, a meta-analysis from 2023, based on 17 clinical trials, concluded that probiotics reduced incidence and progression of dental caries in preschool children [15], which is in concert with another meta-analysis from 2022 [16]. The major discrepancies observed in clinical parameters underscore the necessity to include microbiological endpoints in clinical studies, as knowledge on the microbiological mode of action of probiotics is essential for interpretation of the potential clinical effect observed.

In recent decades, there has been a tremendous development in molecular microbiological methods, which can be used to characterize the oral microbiota [17]. For example, moving from culturing procedures to culture-independent techniques, such as cloning and sequencing some 20 years ago, enabled identification of a substantial part of the uncultured and, therefore hitherto, unknown members of the oral microbiota [18]. Moreover, advancement to high-throughput next-generation sequencing methods facilitated taxonomic characterization of the oral microbiota in health and disease [19]. In continuation, metagenomic sequencing has enabled strain-level taxonomic resolution [20]. Also, advanced methods such as metatranscriptomics and metaproteomics have accelerated the transition from studies on microbial composition toward functional characterization of the oral microbiota [21,22]. Finally, whole-genome sequencing together with metagenome-assembled genomes provide the opportunity for detailed characterization of complete bacterial genomes [23,24]. As such, the molecular toolbox is now heavily equipped with the instruments needed for an in-depth analysis of probiotic strains and their potential impact on the oral microbiota. The question is whether these methods have found their way into the area of probiotics.

The purpose of the present study was to review the literature, with primary emphasis on the microbiological effect of probiotic supplements observed as compared to placebo in randomized clinical trials (RCTs). Secondly, the aim was to evaluate to which extent contemporary molecular methods are employed in clinical trials in the field of oral probiotics.

## 2. Periodontitis

Periodontitis is a multifactorial disease, which is the consequence of an imbalance of the oral microbiota and the host immune system, mediated by compositional changes of the subgingival microbiota and dysregulation of the host response, conditioned by heredity and environmental risk factors such as smoking. The ultimate oral consequence of periodontitis is tooth loss and edentulism, whereas periodontitis is also associated with increased risk of chronic inflammatory conditions such as type 2 diabetes and cardiovascular diseases [3]. Specifically, the subgingival environment in the established periodontal lesion is characterized by microbial dysbiosis, including compositional changes such as a higher abundance of proposed periodontal pathogens, which are predominantly Gram-negative anaerobic rods [25]. Along this line, studies have demonstrated that non-surgical periodontal treatments induce compositional changes to the subgingival microbiota, as illustrated by a higher abundance of Gram-positive rods and cocci in combination with a decrease in abundance of Gram-negative anaerobic bacteria [26,27]. As the most feasible application of probiotics in periodontal treatment is as a supplement to non-surgical treatment, probiotics should ideally augment the effect of this treatment on the subgingival microbiota.

In the last few decades, multiple RCTs with microbiological endpoints have been performed in periodontology ([28,29,30,31,32,33,34,35,36], Table 1), in which probiotic supplements were used either as an adjunct to non-surgical periodontal treatment [28,29,30,32,33,34,35,36], or without additional instrumentation [31]. In most cases, *Lactobacillus* and *Bifidobacterium* species were used as probiotic strains, being delivered in different forms, including mouthwash [28,36], capsules [29], suspensions [31], gels [30], and lozenges [30,32,34,35]. In addition to mode of delivery, there are other significant discrepancies in terms of sample size and study design, with some studies testing the short-term effect after 14 days [28] and others the long-term effect after 12 months [34]. Moreover, different microbiological methods have been used, including culturing, qPCR, and DNA–DNA checkerboard [28,29,30,31,32,33,34,35,36]. Naturally, the heterogenicity observed hampers the possibility of comparing data across the studies included.

From a microbiological point of view, the results seem conflicting, with some studies reporting no effect of the tested probiotic, irrespective of the microbiological endpoint [29,30,31,33,36]. Among the positive effects being reported, these include a significant decrease in *Treponema denticola* and *Tannerella forsythia* in the subgingival plaque as evaluated by PCR [28], a significant decrease in red complex bacteria in the subgingival plaque monitored by DNA–DNA checkerboard [32], a significant decrease in the percentage of obligate anaerobic bacteria in the subgingival plaque identified by culturing [34], and a significant reduction in salivary, supragingival and subgingival levels of *Porphyromonas gingivalis* as quantified by qPCR [35]. While the positive microbiological results can be seen as proof of principle, demonstrating an effect of the tested probiotic in vivo, it is important to remember that periodontitis is a complex disease with a polymicrobial etiology [25]. Hence, a probiotic impact evaluated solely as the effect on one or a few preselected proposed pathogens might not necessarily be of clinical importance.

From a technical perspective, the microbial methods employed for studies on probiotics in RCTs in periodontology are all relatively simple using either culturing techniques or early molecular methods, such as qPCR and DNA–DNA checkerboard. The common denominator of the methods used is that they are all close-ended, meaning that they are targeting a few specific proposed pathogens (culturing and qPCR) and up to a total of 40 pre-selected oral bacterial species (DNA–DNA checkerboard). To the best of our knowledge, no single study examining probiotics in periodontology has used high-throughput open-ended methods, such as 16S sequencing or metagenomics, which would have provided an in-depth characterization of the potential effect of probiotics on the subgingival microbiota, as expressed by alpha and beta diversity, as well as compositional changes. In addition, contemporary sophisticated methods, including metatranscriptomics and metaproteomics, have not been employed, which means that the impact of probiotics on the phenotypic profile of the subgingival microbiota in terms of functional information, such as gene expression, remains unknown. In the last decade, advanced molecular methods have found their way into studies on the general microbiology of periodontitis [19,37], providing detailed insight into the etiological role of the subgingival microbiota in health and disease. Importantly, the current perception of the role of the subgingival microbiota in the pathogenesis of periodontitis has greatly moved from a narrow focus on specific bacterial species toward a more comprehensive view on the total biofilm community, including synergistic and antagonistic interactions between members of the biofilm and interactions with the human host in different ecological conditions [25]. Preferably, future probiotic studies in periodontology with microbial endpoints should employ state-of-the-art molecular methods, providing detailed compositional and functional effects mediated by the probiotic tested.

## 3. Dental Caries

Dental caries is a complex disease, which in essence is the biochemical consequence of prolonged microbial carbohydrate metabolism, resulting in continuous pH drops in mature dental biofilms, facilitated by frequent exposure to dietary sugars [4,38]. Historically, the prime microbial focus in the field of dental caries has been on specific oral bacterial species with proficient carbohydrate metabolism, such as oral streptococci [39] and *Lactobacillus* species [40], with special emphasis on *Streptococcus mutans* due to the versatile armamentarium of caries-associated virulence factors [41]. In addition, studies have reported a positive correlation of salivary levels of *S. mutans* with caries experience [42], and salivary carriage of *S. mutans* has been suggested as a risk factor of future caries activity [43]. From a clinical perspective, probiotics could have multiple areas of application in the field of dental caries, but most importantly it would be suitable for non-invasive treatment of non-cavitated lesions, as well as in the prevention of the development of new lesions.

In the field of dental caries, a substantial number of RCTs have been performed testing the microbiological effect of probiotics in both children and adult populations ([44,45,46,47,48,49,50,51,52,53,54,55,56,57,58,59,60,61], Table 1). As is the case in periodontology, the probiotic strains used in cariology are almost exclusively *Lactobacillus* and *Bifidobacterium* species, being delivered in various ways, including mouthwash [44], yoghurt [45,46], milk [50,51,52,53,58,60,61], tablets [47,48,56], ice cream [49,59], oil [54], and cereals [55]. There are considerable differences in the RCTs with regard to study designs, with some studies evaluating the short-term effect after 7–14 days of consumption [44,45,47,51,60], and others the impact of long-term consumption between 6 and 9 months [50,52,61]. In addition, two studies have monitored the effect of consumption of probiotics during the first year after birth, in 9-year-olds [54,55]. In a substantial amount of these studies, culturing of *S. mutans* and/or *Lactobacillus* species from supragingival plaque and/or saliva samples was the only microbial analysis performed. Likewise, several studies have used chairside detection of *S. mutans* and/or *Lactobacillus* species. While very similar microbial endpoints provide the option for comparison across studies, these will obviously be heavily influenced by differences in study design, delivery modes and composition of cohorts.

In adults, a positive effect of probiotics, as evaluated by a significant decrease in *S. mutans* and/or *Lactobacillus* species in supragingival plaque and/or saliva samples, has been reported in multiple studies [19,44,45,46,47]. Importantly, studies reporting a positive microbiological effect in adults tested the short-term effect of the probiotics, as these were used for 14 days, with the microbial effect being evaluated after 14–30 days. In children and adolescents, the results are more diverging, with some studies reporting a positive effect of probiotic consumption on supragingival and salivary levels of *S. mutans* and/or *Lactobacillus* species [44,50,51,52,53,58,59,61], with other studies reporting no effect of the tested probiotic [54,55,56,57,60]. Notably, a common feature in studies reporting a positive effect was the evaluation of the probiotic effect immediately after short-term (7–14 days) [51,58,59], intermediate (3 months) [53] and long-term (6–9 months) [50,52] consumption of probiotics. In contrast, most studies showing no effect performed microbial evaluation several months to years after having stopped consuming the probiotic compound [54,55,56,57].

Collectively, studies which evaluated the microbial effect of probiotics immediately after a short, intermediate, or long-term consumption in children and adults were able to demonstrate an impact on supragingival and salivary levels of *S. mutans* and/or *Lactobacillus* species, which suggests that probiotic strains, including *Lactobacillus* and *Bifidobacterium* species, have a potential short-term impact on oral levels of proposed caries pathogens in the period of consumption. On the other hand, a persisting effect as evaluated months to years after consumption could not be detected. Hence, microbial data point towards the fact that prolonged consumption is needed to sustain a microbial effect of probiotics in the context of dental caries.

From a technical perspective, it is conspicuous that studies on the microbial effect of probiotics in the context of dental caries are based almost solely on culturing methods targeting proposed caries pathogens such as *S. mutans* and *Lactobacillus* species. Importantly, epidemiological studies have reported that while colonization with *S. mutans* is associated with increased risk of dental caries, *S. mutans* is not detected in a substantial part of dental caries cavities [62,63,64]. In addition, recent studies using contemporary molecular methods have demonstrated taxonomic and functional differences between the supragingival and salivary microbiota in dental caries versus oral health, which is not limited to *S. mutans* and *Lactobacillus* species [65,66,67]. Along this line, studies have demonstrated that other members of the oral microbiota, such as *Veillonella* species and *Streptococcus sobrinus*, may be better predictors of dental caries than *S. mutans* and *Lactobacillus* species [68,69]. Consequently, future probiotic studies in the field of dental caries that are performed using contemporary molecular methods are urgently needed, which will enable a shift in analysis towards focusing on taxonomic and functional characterization of the oral microbiota instead of the hitherto narrow focus on *S. mutans* and *Lactobacillus* species.

## 4. Gingivitis

Gingivitis is the most prevalent oral disease [70], with the microbial component as the central act in the pathogenesis of gingivitis being known since the 1960s [71]. Gingivitis, which is the consequence of undisturbed supragingival biofilm formation and maturation, is considered the predecessor of periodontitis [3], but not all cases of gingivitis will progress to periodontitis [72]. Due to its strong microbial etiology, prevention and treatment of gingivitis, i.e., professional dental cleaning, focus on supra- and subgingival plaque control. Hence, probiotics could be used to augment the microbiological effect of professional dental cleaning in the treatment of gingivitis.

Few RCTs have tested the microbiological effect of probiotics in the treatment of gingivitis ([73,74,75,76,77], Table 1). In gingivitis, different *Lactobacillus* species, including *L. rhamnosus*, *L. curvatus*, *L. plantarum*, *L. brevis*, and *L. reuteri*, have been delivered as tablets [74,75,76,77] or lozenges [73], either during experimental gingivitis [73,76] or as treatment of established gingivitis [74,75,77]. The microbiological effect has been evaluated in supragingival plaque [73,76], saliva [74], subgingival plaque [75], and simultaneously in subgingival plaque and saliva samples [77], using 16S sequencing [73,74], qPCR [75,77], and DNA–DNA checkerboard [76] immediately after probiotic consumption for 28 days to 8 weeks. In general, the comparable study designs, the almost similar study cohorts, as well as less heterogenicity in terms of delivery mode and duration of probiotic intake, assisted comparison of data across studies, while the use of different molecular methods together with different microbial samples being analyzed hampered comparison of data.

Microbiologically, some studies have reported the positive effects of probiotics on the microbial endpoints tested, including microbial resilience to experimental gingivitis in supragingival plaque [73], a significant reduction in subgingival levels of *T. forsythia* [75], and a significant reduction in *P. gingivalis* in subgingival plaque together with a significant reduction in total anaerobic counts and *Prevotella intermedia* in saliva [77]. In one study, no effect was observed on the composition of the salivary microbiota as evaluated by 16S sequencing [74], whereas another study failed to identify any effect on the supragingival microbiota during experimental gingivitis based on DNA–DNA checkerboard analysis [76].

As compared to research on probiotics in periodontology and cariology, two studies have employed modern high-throughput molecular methods for characterization of the salivary and the supragingival microbiota in gingivitis [73,74]. Hence, more detailed knowledge is available on the effect of these probiotic strains in the context of gingivitis, as compared to what could have been retrieved by culturing or use of close-ended methods targeting a limited number of pre-selected species. In addition, the use of 16S provided the opportunity to characterize the effect of the probiotics as evaluated by microbial diversities and relative abundances. Yet, sophisticated methods, such as metatranscriptomics and metaproteomics, which enable focus on bacterial functions and metabolic activity, rather than taxonomic composition, have not been used. Interestingly, a recent study demonstrated that virulence-related genes were upregulated in the transition from oral health to gingivitis, and that these changes were mediated by individual expression by specific bacterial species, underscoring the complexity of biofilm adaptation to the ecological changes accompanying the transition from health to gingivitis [78]. Along this line, two recent studies have reported different clinical trajectories of experimental gingivitis, which is not explained by the magnitude of clinical biofilm formation [79,80]. Taking these findings together, it is important that future studies testing the impact of probiotics on experimental gingivitis stratify and analyze the effect of the probiotic strains in individuals with different response patterns to experimental gingivitis, and subsequently use advanced molecular methods to illuminate bacterial gene expression inflicted by the probiotic tested.

## 5. Oral Health

The oral microbiota is the second most complex found in the human organism [81], with studies showing that the oral microbiota expresses both short- and long-term compositional stability if the ecological balance of the oral cavity is not disturbed [82,83]. On the contrary, external perturbations such as inadequate oral hygiene [84], frequent sugar intake [85], and use of systemic antibiotics [86] rapidly induce compositional changes to the oral microbiota. Hence, from a preventive perspective, if probiotics are to be used by orally healthy individuals, the aim should be to support compositional stability and resilience of the oral microbiota, when faced with stressful conditions.

Several probiotic RCTs with a microbial endpoint have been performed in orally healthy individuals ([87,88,89,90,91,92,93,94], Table 1), testing *Lactobacillus*, *Bifidobacterium*, and *Streptococcus* strains delivered as either tablets [91,92,93,94], lozenges [87,88,90] or gel [89] after consumption for 4–12 weeks. Microbial evaluation was performed immediately in either supragingival plaque, saliva, or subgingival plaque by means of different molecular methods, including 16S sequencing [87,88,89], qPCR [90,91,94], Human Oral Microbe Identification Microarray (HOMIM) [92], and DNA–DNA checkerboard [93]. The comparable study designs and the similar study cohorts together with the immediate evaluation of microbial endpoints facilitated the comparison of data, with different microbial identification methods and the use of various probiotic strains being the main confounding factors.

**Table 1:** Probiotic randomized clinical trials in periodontitis, dental caries, gingivitis and oral health.

Author, Year, Reference	Country	Sample Size	Probiotic Strains	Delivery Mode	Microbial Sampling and Analysis	Authors Reported Results
Periodontitis						
Tapashetti et al., 2022, [28]	India	N = 20	*Lactobacillus acidophilus* *Lactobacillus rhamnosus* *Bifidobacterium longum* *Saccharomyces boulardii*	Mouthwash 2 times per day, 14 days	qPCR subgingival plaque	Significant decrease in *Treponema denticola* and *Tannerella forsythia*
De Oliveira et al., 2022, [29]	Brazil	N = 48	3 *Lactobacillus* spp. 2 *Bifidobacterium* spp.	Capsule 1 capsule per day, 30 days	DNA–DNA checkerboard Subgingival plaque	No significant changes
Pudgar et al., 2021, [30]	Slovenia	N = 40	*Lactobacillus brevis* *Lactobacillus plantarum*	Gel and lozenges 1 time per day, 3 months	Culturing Subgingival plaque	No significant changes
NĘdzi-GÓra et al., 2020, [31]	Poland	N = 51	*Lactobacillus salivarius* SGL03	Suspension 1 time per day, 30 days	Culturing Supragingival plaque	No significant changes
Invernici et al., 2018, [32]	Brazil	N = 41	*Bifidobacterium animalis* subsp. lactis (*B. lactis*) HN019	Lozenges 1 time per day, 30 days	DNA–DNA checkerboard Subgingival plaque	Significant decrease in red complex bacteria
Morales et al., 2018, [33]	Chile	N = 47	*Lactobacillus rhamnosus* SP1	Sachet 1 time per day, 3 months	DNA–DNA checkerboard Culturing Subgingival plaque	No significant changes
Tekce et al., 2015, [34]	Turkey	N = 40	*Lactobacillus reuteri*	Lozenges 2 times per day, 3 weeks	Culturing Subgingival plaque	Significant decrease in % of obligate anaerobes
Teughels et al., 2013, [35]	Belgium	N = 30	*Lactobacillus reuteri*	Lozenges 2 times per day, 12 weeks	qPCR Saliva, supragingival and subgingival plaque	Significant decrease in *Porphyromonas gingivalis* in saliva, supragingival and subgingival plaque
Tsubaru et al., 2009, [36]	Japan	N = 54	*Bacillus subtilis*	Mouthwash 2 times per day, 1 month	BANA test/hybridization Supragingival plaque	No significant changes
Dental Caries in adults						
Krupa et al., 2022, [44]	India	N = 30	*Lactobacillus acidophilus*-R 0052 *Lactobacillus rhamnosus*-R 0011 *Bifidobacterium longum*-R 00175 *Bacillus coagulans*-SNZ 1969 *Saccharomyces boulardii*	Mouthwash 2 times per day, 14 days	Culturing Supragingival plaque	Significant decrease in *Streptococcus mutans*
Javid et al., 2020, [45]	Iran	N = 66	*Bifidobacterium lactis* Bb12	Yoghurt 1 time per day, 14 days	Culturing Saliva	Significant decrease in *Streptococcus mutans* and *Lactobacillus* spp.
Ghamesi et al., 2017, [46]	Iran	N = 50	*Lactobacillus acidophilus*	Yoghurt 1 time per day, 3 weeks	Culturing Saliva	Significant decrease in *Streptococcus mutans*
Nishihara et al., 2014, [47]	Japan	N = 64	*Lactobacillus salivarius* WB21 *Lactobacillus salivarius* TI 2711	Tablets 3 times per day, 14 days	Culturing Saliva	Significant decrease in *Streptococcus mutans*
Chuang et al., 2011, [48]	China	N = 78	*Lactobacillus paracasei* GMNL-33	Tablets 3 timers per day, 14 days	SM and LB strip Saliva	No significant changes
Caglar et al., 2008, [49]	Turkey	N = 24	*Bifidobacterium animalis* subsp. lactis BB-12	Ice cream 1 time per day, 10 days	SM and LB strip Saliva	Significant decrease in *Streptococcus mutans*
Dental Caries in children/adolescents						
Krupa et al., 2022, [44]	India	N = 30	*Lactobacillus acidophilus*-R 0052 *Lactobacillus rhamnosus*-R 0011 *Bifidobacterium longum*-R 00175 *Bacillus coagulans*-SNZ 1969 *Saccharomyces boulardii*	Mouthwash 2 times per day, 14 days	Culturing Supragingival plaque	Significant decrease in *Streptococcus mutans*
Manmontri et al., 2020, [50]	Thailand	N = 487	*Lactobacillus paracasei*	Milk 3 times per week, 6 months	qPCR Culturing Saliva, supragingival plaque	Significant decrease in *Streptococcus mutans* and *Lactobacillus* spp.
Patil et al., 2019, [51]	India	N = 30	*Lactobacillus casei*	Milk 1 time per day, 7 days	Culturing Saliva	Significant decrease in *Streptococcus mutans*
Villavicencio et al., 2018, [52]	Colombia	N = 363	*Lactobacillus rhamnosus* *Bifidobacterim longum*	Milk 5 days per week, 9 months	Culturing Saliva	Significant decrease in *Lactobacillus* spp.
Pahumunto et al., 2018, [53]	Thailand	N = 124	*Lactobacillus paracasei* SD1	Milk 1 time per day, 3 months	Culturing Saliva	Significant decrease in *Streptococcus mutans*
Stensson et al., 2014, [54]	Sweden	N = 113	*Lactobacillus reuteri* strain ATCC 55370	Oil 5 drops per day, 1 year	Culturing Saliva, supragingival plaque	No significant changes
Hasslöf et al., 2013, [55]	Sweden	N = 179	*Lactobacillus paracasei* F19	Cereals 1 time per day, 9 months	Culturing Saliva	No significant changes
Taipale et al., 2013, [56]	Finland	N = 163	*Bifidobacterium animalis* subsp. lactis BB-12	Tablets 1 time per day, 2 years	Culturing SM strip Supragingival plaque	No significant changes
Burton et al., 2013, [57]	New Zealand	N = 100	*Streptococcus salivarius* M18	Lozenges 2 times per day, 3 months	Culturing Saliva	No significant changes
Juneja et al., 2012, [58]	India	N = 40	*Lactobacillus rhamnosus* hct 70	Milk 1 time per day, 3 weeks	Culturing Saliva	Significant decrease in *Streptococcus mutans*
Singh et al., 2011, [59]	India	N = 40	*Bifidobacterium lactis* Bb12 *Lactobacillus acidophilus* La5	Ice cream 1 time per day, 10 days	SM and LB strip Saliva	Significant decrease in *Streptococcus mutans*
Lexner et al., 2010, [60]	Denmark	N = 18	*Lactobacillus rhamnosus* LB21	Milk 1 time per day, 14 days	DNA–DNA checkerboard Culturing Saliva	No significant changes
Näse et al., 2001, [61]	Finland	N = 594	*Lactobacillus rhamnosus* GG	Milk 5 days per week, 7 months	Culturing Saliva, supragingival plaque	Significant decrease in *Streptococcus mutans*
Gingivitis						
Lundtorp Olsen et al., 2023, [73]	Denmark	N = 80	*Lactobacillus rhamnosus* PB01, DSM 14869 *Lactobacillus curvatus* EB10, DSM 3230	Lozenges 2 times per day, 28 days	16S sequencing Supragingival plaque	Significant impact on resilience of the supragingival microbiota
Keller et al., 2018, [74]	Denmark	N = 47	*Lactobacillus rhamnosus* PB01, DSM 14869 *Lactobacillus curvatus* EB10, DSM 3230	Tablets 2 times per day, 28 days	16S sequencing Saliva	No significant changes
Montero et al., 2017, [75]	Spain	N = 59	*Lactobacillus plantarum*, *Lactobacillus brevis* and *Pediococcus acidilactici*	Tablets 2 times per day, 6 weeks	qPCR Subgingival plaque	Significant decrease in *Tannerella forsythia*
Hallström et al., 2013, [76]	Sweden	N = 18	*Lactobacillus reuteri* (ATCC55730 and ATCC PTA5289)	Tablets 2 times per day, 3 weeks	DNA–DNA checkerboard Supragingival plaque	No significant changes
Iniesta et at., 2012, [77]	Spain	N = 40	*Lactobacillus reuteri*	Tablets 1 time per day, 8 weeks	qPCR Culturing	Significant decrease in *Porphyromonas gingivalis* and *Prevotella intermedia*
Oral Health						
Lundtorp Olsen et al., 2021, [87]	Denmark	N = 110	*Lactobacillus rhamnosus* PB01, DSM 14869 *Lactobacillus curvatus* EB10, DSM 3230	Lozenges 2 times per day, 12 weeks	16S sequencing Supragingival plaque	No significant changes
Lundtorp Olsen et al., 2021, [88]	Denmark	N = 80	*Lactobacillus rhamnosus* PB01, DSM 14869 *Lactobacillus curvatus* EB10, DSM 3230	Lozenges 2 times per day, 28 days	16S sequencing Saliva	Significant decrease in *Streptococcus* spp.
Ferrer et al., 2020, [89]	Spain	N = 59	*Streptococcus dentisani* 7746	Gel 1 time per day, 1 months	16S sequencing Supragingival plaque	Significant change in microbiota composition
Alanzi et al., 2018, [90]	Kuwait	N = 108	*Lactobacillus rhamnosus* GG (LGG) *Bifidobacterium lactis* BB-12	Lozenges 2 times per day, 4 weeks	qPCR Saliva, supragingival plaque	Significant decrease in *Aggregatibacter actinomycetemcomitans*, *Porphyromonas gingivalis* and *Fosubacterium nucleatum*
Tobia et al., 2018, [91]	Japan	N = 16	*Lactobacillus crispatus* KT-11 strain (KT-11)	Tablets 1 time per day, 4 weeks	qPCR Saliva	Significant decrease in *Porphyromonas gingivalis*
Toiviainen et al., 2015, [92]	Finland	N = 60	*Lactobacillus rhamnosus* GG *Bifidobacterium animalis* subsp. lactis BB-12	Tablets 1 time per day, 4 weeks	HOMIM Culturing Saliva	No significant changes
Sinkiewitz, et al., 2010, [93]	Sweden	N = 23	*Lactobacillus reuteri* ATCC 55730 and ATCC PTA 5289	Tablets 1 time per day, 12 weeks	DNA–DNA checkerboard Culturing Saliva	No significant changes
Mayanagi et al., 2009, [94]	Japan	N = 66	*Lactobacillus salivarius* WB21	Tablets 1 time per day, 4 weeks	qPCR Supragingival plaque	Significant decrease in periopathogens

Microbiologically, a handful of studies have reported a positive effect of the probiotic tested, such as significant compositional changes in the supragingival and salivary microbiota as characterized by 16S sequencing [88,89], and significant reductions in the proposed periodontal pathogens in plaque and saliva quantified by qPCR [90,91,94]. On the contrary, other studies failed to show an effect on supragingival plaque and saliva composition and levels of selected species as evaluated by means of 16S sequencing [87], HOMIM and culturing [93] and qPCR [94].

From a biological perspective, it is noteworthy that a considerable number of clinical trials performed in orally healthy individuals have focused primarily on the supragingival and salivary levels of proposed periodontal pathogens, when considering that salivary and supragingival carriage of these specific species is reported as relatively low in healthy adults [95,96,97,98]. Hence, one could argue that specific pathogenic species are not the most appropriate target for probiotics used in orally healthy individuals. In continuation, in two studies, where high-throughput sequencing demonstrated a significant impact of probiotics on the salivary [88] and the supragingival microbiota [89], the compositional changes were primarily driven by alterations in relative abundance of *Streptococcus* species in saliva, and supragingival abundance of the proposed cariogenic pathogen, *Scardovia wiggsiae*. While high-throughput molecular methods have already been used to study the effect of probiotics on the healthy oral microbiome, the working mechanisms remain to be uncovered, as no studies have employed methods which enable functional characterization of the microbiota.

## 6. Discussion

**Figure 1:**
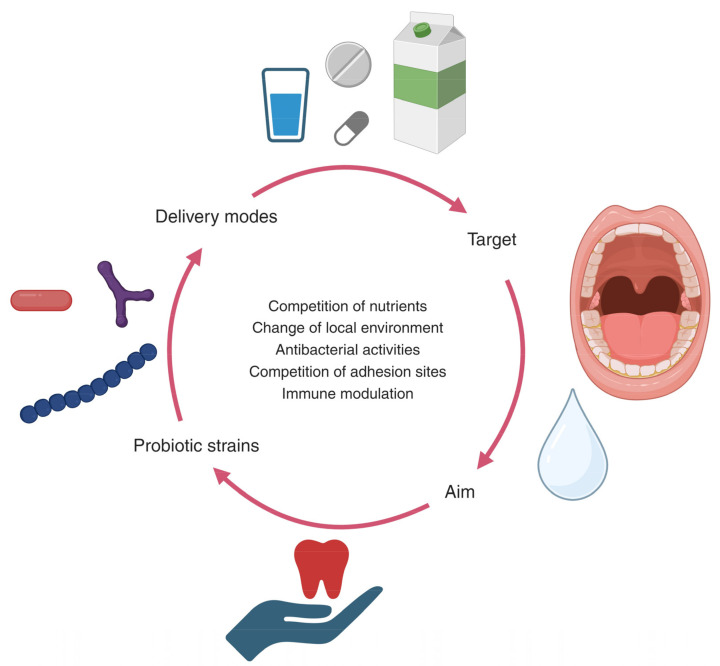
Oral probiotics aim to interact with the host microbiome to support oral health and halt the progression of oral diseases.

The present review of the literature has identified significant microbiological shortcomings in the research area of probiotics, as most RCTs do not have a microbiological endpoint. Indeed, this is a concern when considering that, from a theoretical perspective, some of the main proposed working mechanisms of probiotics, irrespective of body site, are direct and indirect interactions with the resident microbiota ([99,100], Figure 1). Arguably, microbiological data are therefore essential when interpreting clinical endpoints in clinical probiotic trials.

From a legislative point of view, probiotics are categorized as food supplements, which means that the extensive battery of rules and regulations from the pharmaceutical area does not apply to the probiotics industry. In other words, it is possible to produce and sell probiotics without having provided data showing the safety and clinical efficacy of the product. To the best of our knowledge, only one study has been conducted aimed specifically at testing the clinical and microbiological safety of a probiotic compound [87]. Certainly, this is surprising, considering that the global probiotics market is estimated to reach USD 85.4 billion in 2027 (https://www.marketsandmarkets.com/, accessed on 14 March 2024), meaning that it should be financially possible to thoroughly test probiotic products before being released to the market. Importantly, the effects on the oral microbiota of other oral care products, such as toothpaste and dentifrices, have been analyzed by means of advanced molecular methods, despite the fact that these products, like probiotics, are also not categorized as medical compounds [101,102]. Naturally, it is a great advantage that solid evidence is available, assisting dental professionals when they advise their patients which oral health care products to use, including probiotics.

Technically, the first step of screening for probiotic strains is performed using in vitro laboratory analysis, focusing on the effect of the probiotics on specific predefined microbial pathogens [103,104], which might explain why most probiotic RCTs solely investigate oral levels of specific predefined pathogens. While an effect observed in the laboratory is a prerequisite for further analysis, it is important to acknowledge that data generated using culturing and other in vitro setups represent a simplified version, as compared to the in vivo condition, where the probiotic will be in competition with the resident microbiota and influenced by the host. Hence, it is pivotal to address the impact of the probiotic not only on the preselected pathogens, but also on the total microbial community, requiring more sophisticated methods than culturing, qPCR, and DNA–DNA checkerboard, which until now are the techniques predominantly used for studies on probiotics in RCTs.

From a molecular perspective, oral health may be composed of different microbial and metabolomic profiles [105]. In addition, the composition of the oral microbiota is highly site-specific [106], and influenced not only by oral health status, but also by general medical disorders and age [107,108,109]. Moreover, frequency and mode of delivery are of critical importance, as probiotic supplements will most likely have the most pronounced effect in situations where the oral biofilm is also being mechanically disrupted. In addition, the probiotic supplement should ideally be present in the oral cavity for a prolonged time to have maximal effect. Hence, the ideal frequency and mode of delivery will most likely not be the same in the context of dental caries, gingivitis, and periodontitis. Consequently, it is critically important when choosing a probiotic to counterweigh the expected beneficial effects at diseased sites against potential adverse effects at other oral sites, or in predisposed individuals. With that in mind, it is staggering that almost identical probiotic strains have been tested in both periodontitis and caries, when considering that proposed pathogens of the two diseases are critically different in terms of their ecological preferences (pH and O2) and metabolic profile [110]. Hence, from a theoretical point of view, caries probiotics could potentially favor periodontal pathogens and vice versa. Importantly, epidemiological evidence suggests different individual predispositions to the development of dental caries and periodontitis [111], which is in line with recent data on experimental gingivitis, showing different inflammatory reaction patterns to biofilm formation [79,80]. Hence, from a biological perspective, we speculate that the same probiotic strain could have different microbiological effects based on parameters such as baseline microbial composition, age, gender, as well as oral and general health status. Accordingly, this call for action, with various probiotics being used in individuals with different oral health risk profiles, is part of an individualized oral precision medicine strategy, as known from other areas such as oncology [112].

A substantial number of probiotic studies focus on the abundance of specific predefined pathogens, such as *S. mutans* and *P. gingivalis*, thereby adhering to key elements of the specific plaque hypothesis, which was rejected in the mid-1990s and substituted by the ecological plaque hypothesis [113]. As initiated by the red complex theory [114], and further developed by the keystone pathogen hypothesis [115], *P. gingivalis* has attracted considerable attention as an etiological agent of periodontitis, which is biologically grounded, as *P. gingivalis* possesses a wide variety of periodontitis-associated virulence factors [116]. While the former perception of *P. gingivalis* was that virulence was primarily the consequence of high subgingival abundance, recent literature using state-of-the-art molecular methods points towards *P. gingivalis* being highly pathogenic even in low numbers, as the pathogenicity is mediated through interactions with the resident microbiota and the human complement system [117,118]. Consequently, *P. gingivalis* can potentially still orchestrate prolonged disease activity, despite being deprived in number by a probiotic. However, this will not be identified using simple molecular methods focusing solely on levels of *P. gingivalis* or other specific bacteria. Interestingly, recent literature has employed metatranscriptomics to portray in detail microbial activity in periodontitis, as quantified not only by bacterial gene expression of *P. gingivalis* [119], but also resident members of the oral microbiota such as oral streptococci [37]. As such, contemporary data could be used in future development of next-generation probiotics in periodontology, focusing on both depressing pathogenic gene expression and augmenting natural counterbalancing gene expression of the resident oral microbiota.

The focus of the present review is solely on the effect of probiotic supplements on the oral microbiota, which is why studies on prebiotics, synbiotics, and postbiotics were not included. However, it is important to stress that studies have demonstrated the potential of using prebiotics such as arginine and non-cariogenic sugars in the prevention of dental caries [120,121], as well as dietary fibers in the prevention of periodontitis [122], thereby illuminating a preventive potential of prebiotics in oral care.

## 7. Concluding Remarks

One of the main expected working mechanisms of probiotics is through direct and indirect interactions with the resident oral microbiota. Yet, most clinical oral probiotic RCTs have not addressed the microbial effect of the probiotic tested. Hence, to facilitate future systematic reviews and meta-analyses, microbial endpoints should ideally be considered mandatory in all probiotic clinical trials. In addition, an internationally agreed best practice guideline on clinical trials on oral probiotics should be developed by the probiotic scientific community, inspired by important guidelines such as the STROBE guidelines [123] and the PRISMA guidelines [124]. Setting an international standard in terms of study design, with time of delivery as well as mode of delivery depending on the clinical condition, as well as core outcomes for the reporting of microbial endpoints in clinical trials, would be desirable, as such a standardized international guideline would most likely improve the quality of probiotic research in the oral context.
